# Chloridobis(ethyl­enediamine-κ^2^ *N*,*N*′)(*n*-pentyl­amine-κ*N*)cobalt(III) dichloride monhydrate

**DOI:** 10.1107/S1600536809022764

**Published:** 2009-06-27

**Authors:** K. Anbalagan, M. Tamilselvan, S. Nirmala, L. Sudha

**Affiliations:** a Department of Chemistry, Pondicherry University, Puducherry 605 014, India; b Department of Physics, Easwari Engineering College, Ramapuram, Chennai 600 089, India; c Department of Physics, SRM University, Ramapuram Campus, Chennai 600089, India.

## Abstract

The title complex, [CoCl(C_5_H_13_N)(C_2_H_8_N_2_)_2_]Cl_2_·H_2_O, comprises one chloridobis(ethyl­enediamine)(*n*-pentyl­amine)cobalt(III) cation, two chloride counter-anions and a water mol­ecule. The Co^III^ atom of the complex is hexa­coordinated by five N and one Cl atoms. The five N atoms are from two chelating ethyl­enediamine and one *n*-pentyl­amine ligands. Neighbouring cations and anions are connected by N—H⋯Cl and N—H⋯O hydrogen bonds to each other and also to the water mol­ecule.

## Related literature

For the potential applications of metal–chelate complexes, see: Tweedy (1964 ▶); Kralova *et al.* (2004 ▶); Parekh *et al.* (2005 ▶); Rajevel *et al.* (2008 ▶). For cobalt(III) complexes, see: Bailer & Clapp (1945 ▶); Bailer & Rollinson (1946 ▶). For a related structure, see: Ou *et al.* (2007 ▶).
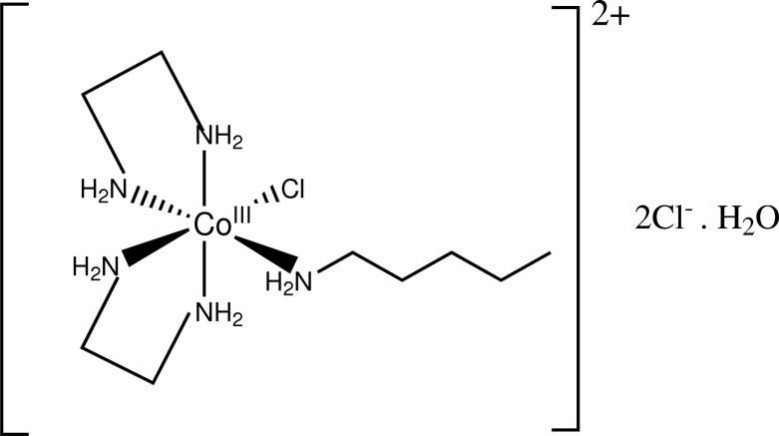


## Experimental

### 

#### Crystal data

[CoCl(C_5_H_13_N)(C_2_H_8_N_2_)_2_]Cl_2_·H_2_O
*M* *_r_* = 390.67
Monoclinic, 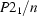

*a* = 10.5214 (3) Å
*b* = 7.2294 (2) Å
*c* = 23.6225 (6) Å
β = 96.117 (2)°
*V* = 1786.58 (8) Å^3^
*Z* = 4
Mo *K*α radiation
μ = 1.41 mm^−1^
*T* = 293 K
0.25 × 0.20 × 0.15 mm

#### Data collection

Bruker Kappa-APEX2 CCD diffractometer
Absorption correction: multi-scan (Blessing, 1995 ▶) *T*_min_ = 0.719, *T*_max_ = 0.816
23262 measured reflections
5510 independent reflections
4506 reflections with *I* > 2σ(*I*)
*R*_int_ = 0.029

#### Refinement

*R*[*F* ^2^ > 2σ(*F* ^2^)] = 0.030
*wR*(*F* ^2^) = 0.090
*S* = 1.10
5510 reflections
181 parameters
3 restraints
H atoms treated by a mixture of independent and constrained refinement
Δρ_max_ = 0.52 e Å^−3^
Δρ_min_ = −0.35 e Å^−3^

### 

Data collection: *APEX2* (Bruker, 2004 ▶); cell refinement: *APEX2* and *SAINT* (Bruker, 2004 ▶); data reduction: *SAINT* and *XPREP* (Bruker, 2004 ▶); program(s) used to solve structure: *SIR92* (Altomare *et al.*, 1993 ▶); program(s) used to refine structure: *SHELXL97* (Sheldrick, 2008 ▶); molecular graphics: *ORTEP-3* (Farrugia, 1997 ▶); software used to prepare material for publication: *PLATON* (Spek, 2009 ▶).

## Supplementary Material

Crystal structure: contains datablocks I, global. DOI: 10.1107/S1600536809022764/bq2142sup1.cif

Structure factors: contains datablocks I. DOI: 10.1107/S1600536809022764/bq2142Isup2.hkl

Additional supplementary materials:  crystallographic information; 3D view; checkCIF report

## Figures and Tables

**Table 1:** Hydrogen-bond geometry (Å, °)

*D*—H⋯*A*	*D*—H	H⋯*A*	*D*⋯*A*	*D*—H⋯*A*
N1—H1*C*⋯Cl3	0.90	2.39	3.2589 (14)	162
N1—H1*D*⋯O1	0.90	2.12	3.018 (3)	174
N3—H3*C*⋯Cl3	0.90	2.56	3.3641 (14)	150
N4—H4*D*⋯Cl3^i^	0.90	2.36	3.2597 (14)	179
N4—H4*C*⋯Cl2^ii^	0.90	2.51	3.3731 (15)	161
N5—H5*C*⋯Cl3^iii^	0.90	2.51	3.3605 (15)	158
N5—H5*D*⋯Cl3^i^	0.90	2.56	3.3784 (15)	151

Symmetry codes: (i) 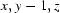; (ii) 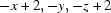; (iii) 
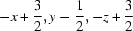
.

**Fig. 1.**
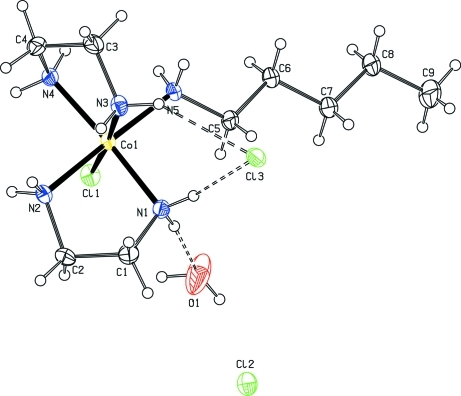
The molecular structure of the title compound with 30% probability displacement ellipsoids. Dashed lines indicate hydrogen bonds.

**Fig. 2.**
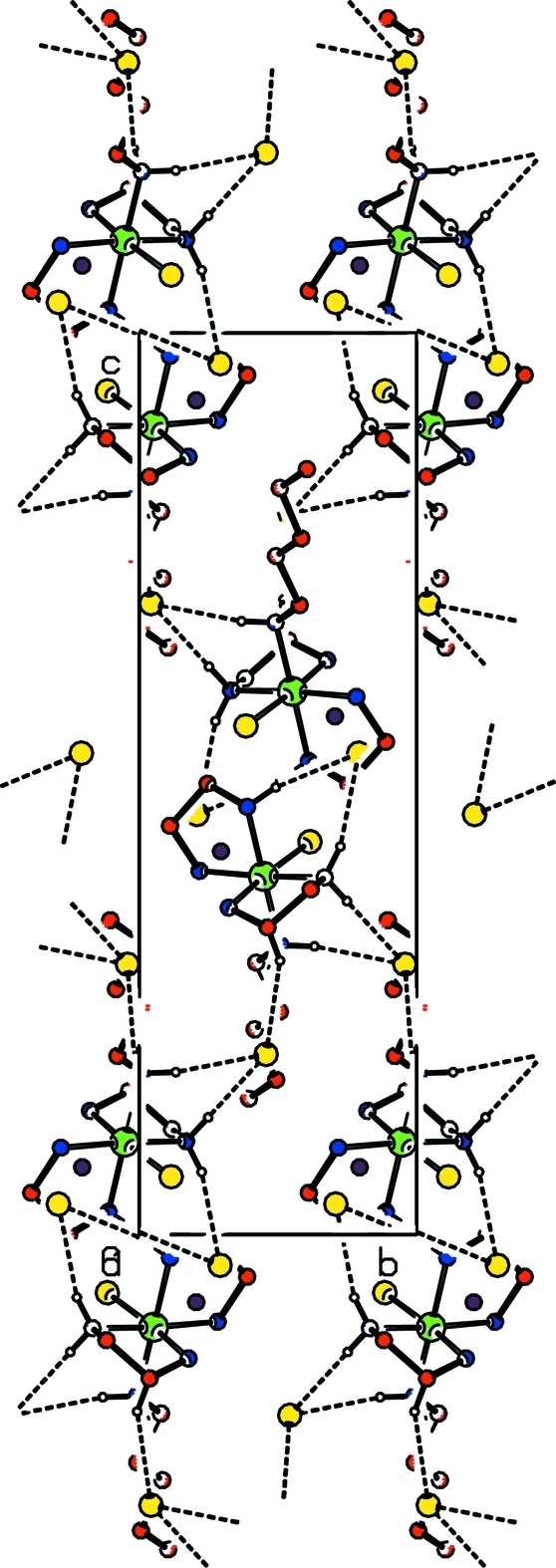
The packing of the molecules viewed down the a axis. Dashed lines indicate hydrogen bonds. H atoms not involved in hydrogen bonds have been omitted.

## supplementary crystallographic information

### Comment

Metal-chelate complexes find potential applications in the research fields (Tweedy, 1964; Kralova *et al.*, 2004) of antitumor activity, enzyme catalysis, functioning of micro organisms and in the respiration processes of biological systems (Parekh *et al.*, 2005; Rajevel *et al.*, 2008). Chelating ligand such as ethylenediamine has been widely used to prepare a number of cobalt(III) complexes (Bailer & Clapp, 1945; Bailer & Rollinson, 1946). A structural analogue of the cobalt(III)-alkyl amine complex, such as chloro(n-pentyl amine)bis(ethylenediamine)cobalt(III) chloride, [Co^III^(en)_2_(nPentNH_2_) Cl]Cl_2_, is studied. Cobalt(III) complex consisting of n-PentNH_2 _ligand, is an interesting complex showing some novel reactivity. Hence, single-crystal X-ray study of the above compound has been carried out.

The molecular structure of the title compound is shown in Fig. 1. The title compound, Cis-[Co^III^(en)_2_(nPentNH_2_)Cl]Cl_2_.H_2_O, is a mononuclear cobalt(III) complex. The Co(III) atom is hexa-coordinated by six ligating atoms (five N and one Cl) forming two chelating ethylenediamine ligands, leading to a slightly distorted octahedral configuration. The two chloride ions act as counter-ions. The average Co— N bond length is 1.963 (4) Å and agrees well with related literature (Ou *et al.*, 2007).

The crystal structure is stabilized by intramolecular N—H···O and N—H···Cl interactions. The molecules are linked into three-dimensional framework by N—H···Cl and C—H···Cl intermolecular interactions (Fig. 2, Table 1).

### Experimental

A modified method of synthesize of *cis*-[Co^III^(en)_2_(nPentNH_2_)Cl]Cl_2_.H_2_O was developed by substituting chloride ligand with AnalaR n-pentyl amine in *trans*-[Co(en)_2_Cl_2_]Cl. AnalaR n-pentyl amine (2–3 ml) was added in drops to a paste of 2 g of the *trans*-dichlorobis(1,2-diamino ethane)cobalt(III) chloride suspended in 1 ml of water. The mixture was ground for an hour until the solid becomes rosy red, and allowed overnight. The complex was recrystallized from acidified water. Single crystal was grown by adding the metal complex in triply distilled water containing few drops of conc. HCl and kept at 0°C for 2–3 weeks.

### Refinement

H atoms were placed in idealized positions and allowed to ride on their parent atoms, with C—H = 0.97Å and 0.96Å for methylene and methyl H respectively, and N—H = 0.86Å, and with *U*_iso_(H) = 1.5Ueq(C) for methyl and *U*_iso_(H) = 1.2Ueq(C,*N*) for all other H atoms. The H atoms of the water molecule were located in a difference Fourier map and their positional parameters refined with *U*_iso_(H) = 1.5Ueq(O), and with the O—H distances restrained to be 0.85 (1)Å.

### Crystal data

**Table:**

[CoCl(C_5_H_13_N)(C_2_H_8_N_2_)_2_]Cl_2_·H_2_O	*F*(000) = 824
*M**_r_* = 390.67	*D*_x_ = 1.452 Mg m^−^^3^
Monoclinic, *P*2_1_/*n*	Mo *K*α radiation, λ = 0.71073 Å
Hall symbol: -P 2yn	Cell parameters from 8809 reflections
*a* = 10.5214 (3) Å	θ = 2.9–30.6°
*b* = 7.2294 (2) Å	µ = 1.41 mm^−^^1^
*c* = 23.6225 (6) Å	*T* = 293 K
β = 96.117 (2)°	Prismatic, orange
*V* = 1786.58 (8) Å^3^	0.25 × 0.20 × 0.15 mm
*Z* = 4	

### Data collection

**Table:**

Bruker Kappa-APEX2 CCD diffractometer	5510 independent reflections
Radiation source: fine-focus sealed tube	4506 reflections with *I* > 2σ(*I*)
graphite	*R*_int_ = 0.029
ω and φ scans	θ_max_ = 30.7°, θ_min_ = 1.7°
Absorption correction: multi-scan (Blessing, 1995)	*h* = −15→15
*T*_min_ = 0.719, *T*_max_ = 0.816	*k* = −8→10
23262 measured reflections	*l* = −33→33

### Refinement

**Table:**

Refinement on *F*^2^	Secondary atom site location: difference Fourier map
Least-squares matrix: full	Hydrogen site location: inferred from neighbouring sites
*R*[*F*^2^ > 2σ(*F*^2^)] = 0.030	H atoms treated by a mixture of independent and constrained refinement
*wR*(*F*^2^) = 0.090	*w* = 1/[σ^2^(*F*_o_^2^) + (0.0497*P*)^2^ + 0.0821*P*] where *P* = (*F*_o_^2^ + 2*F*_c_^2^)/3
*S* = 1.10	(Δ/σ)_max_ = 0.006
5510 reflections	Δρ_max_ = 0.52 e Å^−^^3^
181 parameters	Δρ_min_ = −0.35 e Å^−^^3^
3 restraints	Extinction correction: *SHELXL97* (Sheldrick, 2008), Fc^*^=kFc[1+0.001xFc^2^λ^3^/sin(2θ)]^-1/4^
Primary atom site location: structure-invariant direct methods	Extinction coefficient: 0.0019 (6)

### Special details

**Table:**

Geometry. All e.s.d.'s (except the e.s.d. in the dihedral angle between two l.s. planes) are estimated using the full covariance matrix. The cell e.s.d.'s are taken into account individually in the estimation of e.s.d.'s in distances, angles and torsion angles; correlations between e.s.d.'s in cell parameters are only used when they are defined by crystal symmetry. An approximate (isotropic) treatment of cell e.s.d.'s is used for estimating e.s.d.'s involving l.s. planes.
Refinement. Refinement of *F*^2^ against ALL reflections. The weighted *R*-factor *wR* and goodness of fit *S* are based on *F*^2^, conventional *R*-factors *R* are based on *F*, with *F* set to zero for negative *F*^2^. The threshold expression of *F*^2^ > σ(*F*^2^) is used only for calculating *R*-factors(gt) *etc*. and is not relevant to the choice of reflections for refinement. *R*-factors based on *F*^2^ are statistically about twice as large as those based on *F*, and *R*- factors based on ALL data will be even larger.

### Fractional atomic coordinates and isotropic or equivalent isotropic displacement parameters (Å^2^)

**Table:**

	*x*	*y*	*z*	*U*_iso_*/*U*_eq_	
C1	0.6942 (2)	0.3891 (2)	0.95347 (8)	0.0391 (4)	
H1A	0.6317	0.4750	0.9657	0.047*	
H1B	0.7675	0.4592	0.9439	0.047*	
C2	0.73406 (18)	0.2535 (2)	1.00007 (8)	0.0352 (4)	
H2A	0.7846	0.3145	1.0314	0.042*	
H2B	0.6597	0.1982	1.0143	0.042*	
C3	0.95703 (17)	0.0374 (3)	0.84080 (8)	0.0387 (4)	
H3A	1.0400	0.0913	0.8368	0.046*	
H3B	0.9209	−0.0074	0.8038	0.046*	
C4	0.96996 (17)	−0.1182 (3)	0.88299 (8)	0.0364 (4)	
H4A	1.0107	−0.2237	0.8670	0.044*	
H4B	1.0220	−0.0798	0.9173	0.044*	
C5	0.52205 (16)	0.0781 (2)	0.80180 (8)	0.0328 (4)	
H5A	0.5349	0.2092	0.7957	0.039*	
H5B	0.4656	0.0653	0.8315	0.039*	
C6	0.45868 (16)	−0.0068 (3)	0.74740 (7)	0.0337 (4)	
H6A	0.4451	−0.1377	0.7535	0.040*	
H6B	0.5152	0.0053	0.7177	0.040*	
C7	0.33189 (17)	0.0838 (3)	0.72777 (8)	0.0384 (4)	
H7A	0.3453	0.2155	0.7234	0.046*	
H7B	0.2745	0.0673	0.7569	0.046*	
C8	0.26886 (18)	0.0065 (3)	0.67194 (8)	0.0393 (4)	
H8A	0.3279	0.0169	0.6433	0.047*	
H8B	0.2510	−0.1238	0.6769	0.047*	
C9	0.1464 (2)	0.1043 (4)	0.65112 (12)	0.0659 (7)	
H9A	0.1108	0.0501	0.6158	0.099*	
H9B	0.1636	0.2329	0.6453	0.099*	
H9C	0.0867	0.0922	0.6789	0.099*	
N1	0.63802 (13)	0.28252 (18)	0.90348 (6)	0.0277 (3)	
H1C	0.6409	0.3501	0.8717	0.033*	
H1D	0.5556	0.2567	0.9072	0.033*	
N2	0.81072 (13)	0.11089 (18)	0.97448 (5)	0.0262 (3)	
H2C	0.8144	0.0081	0.9961	0.031*	
H2D	0.8910	0.1525	0.9731	0.031*	
N3	0.87124 (13)	0.1775 (2)	0.86270 (6)	0.0304 (3)	
H3C	0.8378	0.2501	0.8339	0.036*	
H3D	0.9161	0.2495	0.8887	0.036*	
N4	0.84066 (13)	−0.16872 (19)	0.89632 (6)	0.0287 (3)	
H4C	0.8457	−0.2249	0.9305	0.034*	
H4D	0.8052	−0.2485	0.8700	0.034*	
N5	0.64631 (13)	−0.0082 (2)	0.82114 (6)	0.0280 (3)	
H5C	0.7005	0.0201	0.7954	0.034*	
H5D	0.6350	−0.1316	0.8197	0.034*	
O1	0.3655 (2)	0.2049 (5)	0.92521 (12)	0.1255 (12)	
Cl1	0.58524 (4)	−0.11512 (6)	0.935779 (18)	0.03265 (10)	
Cl2	1.09085 (4)	0.29130 (6)	0.96604 (2)	0.03712 (11)	
Cl3	0.71601 (4)	0.54255 (6)	0.799916 (18)	0.03417 (10)	
Co1	0.733516 (18)	0.05224 (3)	0.897435 (8)	0.02096 (7)	
H1E	0.2883 (17)	0.237 (6)	0.9277 (18)	0.17 (2)*	
H1F	0.394 (3)	0.137 (4)	0.9526 (11)	0.112 (13)*	

### Atomic displacement parameters (Å^2^)

**Table:**

	*U*^11^	*U*^22^	*U*^33^	*U*^12^	*U*^13^	*U*^23^
C1	0.0500 (11)	0.0276 (8)	0.0377 (10)	0.0041 (7)	−0.0040 (8)	−0.0063 (7)
C2	0.0430 (10)	0.0363 (9)	0.0258 (8)	0.0007 (7)	0.0007 (7)	−0.0091 (7)
C3	0.0290 (8)	0.0591 (12)	0.0287 (9)	0.0046 (8)	0.0069 (7)	−0.0004 (8)
C4	0.0280 (8)	0.0435 (10)	0.0368 (10)	0.0095 (7)	−0.0005 (7)	−0.0062 (8)
C5	0.0286 (8)	0.0373 (9)	0.0305 (9)	0.0047 (6)	−0.0068 (6)	−0.0044 (7)
C6	0.0306 (8)	0.0408 (9)	0.0279 (9)	0.0023 (7)	−0.0056 (6)	−0.0024 (7)
C7	0.0321 (9)	0.0480 (10)	0.0328 (10)	0.0057 (7)	−0.0068 (7)	−0.0062 (8)
C8	0.0350 (9)	0.0469 (10)	0.0331 (10)	0.0021 (8)	−0.0097 (7)	−0.0036 (8)
C9	0.0468 (13)	0.0812 (17)	0.0632 (16)	0.0152 (12)	−0.0241 (11)	−0.0116 (13)
N1	0.0287 (7)	0.0261 (6)	0.0278 (7)	0.0018 (5)	0.0005 (5)	0.0014 (5)
N2	0.0274 (6)	0.0286 (6)	0.0218 (6)	−0.0027 (5)	−0.0017 (5)	−0.0002 (5)
N3	0.0264 (6)	0.0369 (7)	0.0274 (7)	−0.0017 (5)	0.0017 (5)	0.0070 (6)
N4	0.0313 (7)	0.0283 (6)	0.0250 (7)	0.0035 (5)	−0.0037 (5)	−0.0030 (5)
N5	0.0276 (6)	0.0345 (7)	0.0208 (6)	0.0030 (5)	−0.0032 (5)	−0.0009 (5)
O1	0.0775 (15)	0.188 (3)	0.121 (2)	0.0730 (17)	0.0573 (15)	0.104 (2)
Cl1	0.0302 (2)	0.0344 (2)	0.0332 (2)	−0.00734 (15)	0.00280 (15)	0.00406 (16)
Cl2	0.0307 (2)	0.0406 (2)	0.0387 (2)	−0.00616 (16)	−0.00247 (16)	0.00198 (18)
Cl3	0.0438 (2)	0.0327 (2)	0.0259 (2)	−0.00019 (16)	0.00328 (16)	−0.00105 (15)
Co1	0.02095 (11)	0.02308 (11)	0.01830 (11)	−0.00082 (7)	−0.00051 (7)	0.00072 (7)

### Geometric parameters (Å, °)

**Table:**

C1—N1	1.479 (2)	C8—C9	1.505 (3)
C1—C2	1.501 (3)	C8—H8A	0.9700
C1—H1A	0.9700	C8—H8B	0.9700
C1—H1B	0.9700	C9—H9A	0.9600
C2—N2	1.477 (2)	C9—H9B	0.9600
C2—H2A	0.9700	C9—H9C	0.9600
C2—H2B	0.9700	N1—Co1	1.9575 (13)
C3—N3	1.485 (2)	N1—H1C	0.9000
C3—C4	1.500 (3)	N1—H1D	0.9000
C3—H3A	0.9700	N2—Co1	1.9588 (13)
C3—H3B	0.9700	N2—H2C	0.9000
C4—N4	1.475 (2)	N2—H2D	0.9000
C4—H4A	0.9700	N3—Co1	1.9611 (13)
C4—H4B	0.9700	N3—H3C	0.9000
C5—N5	1.477 (2)	N3—H3D	0.9000
C5—C6	1.513 (2)	N4—Co1	1.9569 (13)
C5—H5A	0.9700	N4—H4C	0.9000
C5—H5B	0.9700	N4—H4D	0.9000
C6—C7	1.514 (2)	N5—Co1	1.9822 (13)
C6—H6A	0.9700	N5—H5C	0.9000
C6—H6B	0.9700	N5—H5D	0.9000
C7—C8	1.518 (2)	O1—H1E	0.852 (10)
C7—H7A	0.9700	O1—H1F	0.841 (10)
C7—H7B	0.9700	Cl1—Co1	2.2403 (4)
			
N1—C1—C2	107.56 (14)	H9A—C9—H9B	109.5
N1—C1—H1A	110.2	C8—C9—H9C	109.5
C2—C1—H1A	110.2	H9A—C9—H9C	109.5
N1—C1—H1B	110.2	H9B—C9—H9C	109.5
C2—C1—H1B	110.2	C1—N1—Co1	109.70 (11)
H1A—C1—H1B	108.5	C1—N1—H1C	109.7
N2—C2—C1	106.15 (14)	Co1—N1—H1C	109.7
N2—C2—H2A	110.5	C1—N1—H1D	109.7
C1—C2—H2A	110.5	Co1—N1—H1D	109.7
N2—C2—H2B	110.5	H1C—N1—H1D	108.2
C1—C2—H2B	110.5	C2—N2—Co1	109.93 (10)
H2A—C2—H2B	108.7	C2—N2—H2C	109.7
N3—C3—C4	107.18 (14)	Co1—N2—H2C	109.7
N3—C3—H3A	110.3	C2—N2—H2D	109.7
C4—C3—H3A	110.3	Co1—N2—H2D	109.7
N3—C3—H3B	110.3	H2C—N2—H2D	108.2
C4—C3—H3B	110.3	C3—N3—Co1	109.55 (11)
H3A—C3—H3B	108.5	C3—N3—H3C	109.8
N4—C4—C3	107.89 (14)	Co1—N3—H3C	109.8
N4—C4—H4A	110.1	C3—N3—H3D	109.8
C3—C4—H4A	110.1	Co1—N3—H3D	109.8
N4—C4—H4B	110.1	H3C—N3—H3D	108.2
C3—C4—H4B	110.1	C4—N4—Co1	110.28 (11)
H4A—C4—H4B	108.4	C4—N4—H4C	109.6
N5—C5—C6	112.74 (14)	Co1—N4—H4C	109.6
N5—C5—H5A	109.0	C4—N4—H4D	109.6
C6—C5—H5A	109.0	Co1—N4—H4D	109.6
N5—C5—H5B	109.0	H4C—N4—H4D	108.1
C6—C5—H5B	109.0	C5—N5—Co1	119.79 (10)
H5A—C5—H5B	107.8	C5—N5—H5C	107.4
C5—C6—C7	112.26 (15)	Co1—N5—H5C	107.4
C5—C6—H6A	109.2	C5—N5—H5D	107.4
C7—C6—H6A	109.2	Co1—N5—H5D	107.4
C5—C6—H6B	109.2	H5C—N5—H5D	106.9
C7—C6—H6B	109.2	H1E—O1—H1F	111.3 (17)
H6A—C6—H6B	107.9	N4—Co1—N1	174.92 (6)
C6—C7—C8	113.34 (15)	N4—Co1—N2	90.39 (6)
C6—C7—H7A	108.9	N1—Co1—N2	85.03 (6)
C8—C7—H7A	108.9	N4—Co1—N3	85.34 (6)
C6—C7—H7B	108.9	N1—Co1—N3	92.63 (6)
C8—C7—H7B	108.9	N2—Co1—N3	92.13 (6)
H7A—C7—H7B	107.7	N4—Co1—N5	91.10 (6)
C9—C8—C7	112.99 (18)	N1—Co1—N5	93.59 (6)
C9—C8—H8A	109.0	N2—Co1—N5	176.89 (6)
C7—C8—H8A	109.0	N3—Co1—N5	90.71 (6)
C9—C8—H8B	109.0	N4—Co1—Cl1	89.52 (4)
C7—C8—H8B	109.0	N1—Co1—Cl1	92.57 (4)
H8A—C8—H8B	107.8	N2—Co1—Cl1	88.79 (4)
C8—C9—H9A	109.5	N3—Co1—Cl1	174.78 (4)
C8—C9—H9B	109.5	N5—Co1—Cl1	88.49 (4)
			
N1—C1—C2—N2	50.28 (19)	C1—N1—Co1—N3	−79.47 (12)
N3—C3—C4—N4	48.28 (19)	C1—N1—Co1—N5	−170.35 (12)
N5—C5—C6—C7	−179.58 (16)	C1—N1—Co1—Cl1	101.00 (11)
C5—C6—C7—C8	177.60 (17)	C2—N2—Co1—N4	−166.25 (11)
C6—C7—C8—C9	−176.9 (2)	C2—N2—Co1—N1	15.94 (11)
C2—C1—N1—Co1	−37.75 (17)	C2—N2—Co1—N3	108.40 (11)
C1—C2—N2—Co1	−40.09 (16)	C2—N2—Co1—Cl1	−76.74 (10)
C4—C3—N3—Co1	−38.51 (17)	C3—N3—Co1—N4	15.27 (11)
C3—C4—N4—Co1	−36.06 (16)	C3—N3—Co1—N1	−169.40 (11)
C6—C5—N5—Co1	−170.27 (12)	C3—N3—Co1—N2	105.48 (11)
C4—N4—Co1—N2	−80.28 (11)	C3—N3—Co1—N5	−75.78 (12)
C4—N4—Co1—N3	11.82 (11)	C5—N5—Co1—N4	161.69 (13)
C4—N4—Co1—N5	102.45 (11)	C5—N5—Co1—N1	−20.28 (13)
C4—N4—Co1—Cl1	−169.07 (11)	C5—N5—Co1—N3	−112.95 (13)
C1—N1—Co1—N2	12.44 (12)	C5—N5—Co1—Cl1	72.21 (12)

### Hydrogen-bond geometry (Å, °)

**Table:**

*D*—H···*A*	*D*—H	H···*A*	*D*···*A*	*D*—H···*A*
N1—H1C···Cl3	0.90	2.39	3.2589 (14)	162
N1—H1D···O1	0.90	2.12	3.018 (3)	174
N3—H3C···Cl3	0.90	2.56	3.3641 (14)	150
N4—H4D···Cl3^i^	0.90	2.36	3.2597 (14)	179
N4—H4C···Cl2^ii^	0.90	2.51	3.3731 (15)	161
N5—H5C···Cl3^iii^	0.90	2.51	3.3605 (15)	158
N5—H5D···Cl3^i^	0.90	2.56	3.3784 (15)	151
C3—H3B···Cl3^iii^	0.97	2.73	3.616 (2)	152

Symmetry codes: (i) *x*, *y*−1, *z*; (ii) −*x*+2, −*y*, −*z*+2; (iii) −*x*+3/2, *y*−1/2, −*z*+3/2.
